# Daily physical activity following unicompartmental knee arthroplasty: A pilot study

**DOI:** 10.1002/jeo2.70048

**Published:** 2024-11-14

**Authors:** Kevin A. Wu, Eric S. Dilbone, David N. Kugelman, Rahul K. Goel, Sean P. Ryan, Samuel S. Wellman, Michael P. Bolognesi, Thorsten M. Seyler

**Affiliations:** ^1^ Department of Orthopaedic Surgery Duke University Medical Center Durham North Carolina USA

**Keywords:** daily activity metrics, partial knee replacement, patient reported outcomes, unicompartmental knee arthroplasty

## Abstract

**Purpose:**

The purpose of this study was to understand how objective measures of daily activity change following unicompartmental knee arthroplasty (UKA). Objective data on post‐operative changes in daily physical activity following UKA are limited, highlighting the need for studies using wearable technologies to provide real‐time assessments of recovery.

**Methods:**

This pilot study included a secondary analysis of a prospective study of 33 consecutive UKA patients, with data collected using an Apple Watch and a digital care management application. Objective metrics, including step count, steadiness, standing duration and performance on the six‐minute walk test, were analyzed at different post‐operative time points. Descriptive statistics and the Wilcoxon signed‐rank test were used for analysis.

**Results:**

Post‐operatively, there was a significant increase in daily step count at 6 weeks (*p* = 0.017), 6 months (*p* < 0.001) and 12 months (*p* = 0.0018). Steadiness improved significantly at 6 months (*p* = 0.049) and 12 months (*p* = 0.039) post‐operatively. Standing duration increased significantly at all the post‐operative time points (*p* < 0.001). Gait speed did not show significant changes post‐operatively. The estimated six‐minute walk test distance improved significantly at 6 months (*p* = 0.027) and 12 months (*p* = 0.031) post‐operatively.

**Conclusion:**

The study findings suggest that UKA improves daily physical activity levels, reflected by enhanced mobility and function. While gait speed did not significantly change, improvements in step count, steadiness, standing duration and the six‐minute walk test distance indicate enhanced functional capacity and endurance post‐operatively. The study highlights the benefits of UKA in improving functional outcomes in patients with knee osteoarthritis. Further research with larger sample sizes and longer follow‐ups is warranted to confirm these findings.

**Levels of Evidence:**

II.

**Abbreviations**
ACL: anterior cruciate ligament
BMI: body mass index
EQ‐5D: EuroQol‐5 Dimension
IRB: institutional review board
KOOS, JR: Knee Injury and Osteoarthritis Outcome Score, Joint Replacement
OA: osteoarthritis
PCL: posterior cruciate ligament
TKA: total knee arthroplasty
UKA: unicompartmental knee arthroplasty

## INTRODUCTION

Osteoarthritis (OA) of the knee is a prevalent source of pain and disability in older adults, significantly impacting quality of life [2, 23, 26, 28]. Unicompartmental knee arthroplasty (UKA) is a surgical option for medial compartment OA that has garnered increasing interest due to favourable long‐term outcomes [4, 11, 15, 17, 18, 30]. It is particularly suitable for patients with isolated anteromedial OA, which can persist for many years before progressing to tricompartmental changes [1]. The slow rate of progression in unreplaced compartments after UKA suggests a potential advantage over total knee arthroplasty (TKA) in terms of preserving function and delaying or preventing further arthritic changes [10].

Despite the benefits of UKA, it remains a contentious procedure, with ongoing debates about its optimal use and outcomes [12]. There is limited objective data on how the daily physical activity levels change following UKA [14]. Advancements in wearable technologies have opened avenues for researchers and surgeons to collect objective physical activity metrics in patients undergoing UKA [7, 29, 31]. These metrics offer a real‐time assessment of post‐operative recovery, providing valuable insights into the progress of functional recovery following UKA.

Knowing how these objective measures change following UKA would allow surgeons to better understand the utility of this procedure and help inform patients about their expected post‐operative recovery [14]. The aim of this pilot study was to prospectively evaluate 33 consecutive UKAs by describing the changes in daily physical activity levels using objective metrics over different operative time points following UKA. We hypothesize that objective measures of physical activity would demonstrate improvements post‐operatively, reflecting enhanced mobility and function in patients undergoing UKA.

## METHODS

### Study design

This study was a secondary analysis of a prospective cohort of patients undergoing UKA within the period between 23 June 2020 and 20 December 2022. The initial study was a prospective study examining the use of a digital care management application (mymobility® Care Management Platform, Zimmer Biomet) for educational support. The current study was a retrospective secondary analysis of the original study data and received approval from the institutional review board (IRB: Pro00115921) before commencement. The original study population comprised individuals aged 18 years and older, who owned an iPhone (iPhone 8 or newer) (Apple Inc) capable of pairing with an Apple Watch (Series 3 or newer) (Apple Inc), and were scheduled for primary knee arthroplasty, and demonstrated the ability to ambulate with minimal assistance before surgery. Exclusion criteria included a history of substance abuse problems, inflammatory arthropathies, or participation in other interventions that could potentially influence outcomes.

Initially, 204 patients scheduled for knee arthroplasty were recruited for the original study. However, 19 patients withdrew from the study before surgery, resulting in a final cohort of 185 patients. Among these, 152 underwent TKA, while the remaining 33 underwent UKA (Table 1). The patients who withdrew from the original study did so because they were no longer interested in the educational component of the digital application. Patients were indicated for UKA if their X‐ray and clinical history demonstrated unicompartmental OA. The final cohort for the current study consisted of 33 patients who underwent UKA, with an average age of 63.39 ± 9.28 years. The cohort included 15 males (45.5%) with a mean body mass index (BMI) of 31.54 ± 4.58 kg/m². Only one patient (3.0%) had a history of diabetes. The majority of patients (*n* = 31; 93.9%) in the included cohort receive the MOTO Medial Partial Knee implant (Medacta International). There were two patients who received the Oxford® Partial Knee (Zimmer Biomet). The patients underwent UKA using traditional manual techniques, without the assistance of robotic systems.

**Table 1:** Characteristics of the study cohort.

Characteristic	*n* = 33
Age, yr (SD)	63.39 (9.28)
BMI, kg/m^2^ (SD)	31.54 (4.58)
Male, *n* (%)	15 (45.5)
Diabetes, *n* (%)	
Yes	1 (3.0)
No	32 (97.0)

Abbreviations: BMI, body mass index; SD, standard deviation.

### Patient‐reported outcome measures

Patient‐reported outcomes were assessed using the Knee Injury and Osteoarthritis Outcome Score, Joint Replacement (KOOS, JR) and EuroQol‐5 Dimension (EQ‐5D) instruments. The KOOS, JR is a validated, shorter version of the KOOS, designed to measure key dimensions of knee health, including pain, stiffness and physical function, specifically in individuals undergoing joint replacement surgery. The score ranges from 0 to 100, with higher scores indicating better knee function and fewer symptoms. The EQ‐5D is a standardized instrument used to measure health‐related quality of life across five dimensions: mobility, self‐care, usual activities, pain/discomfort and anxiety/depression. It also includes a visual analogue scale (VAS) that records the patient's self‐rated health on a scale from 0 (worst imaginable health) to 100 (best imaginable health). Both the KOOS JR and EQ‐5D were administered preoperatively and at multiple post‐operative intervals (1 month, 3 months, 6 months and 1 year) to evaluate the progression of clinical outcomes and overall quality of life following UKA.

### Daily physical metrics

In this study, eligible participants who provided informed consent were provided with an Apple Watch and access to a digital care management application (mymobility® Care Management Platform, Zimmer Biomet). The Apple Watch was worn on the wrist to monitor participants' objective physical activity. The application offered educational materials, exercise guidance and access to a post‐operative at‐home therapy programme. Data on daily activity and gait parameters, including gait speed, step count, standing duration, steadiness (scored on a scale from 0 to 1.0) and performance on the six‐minute walk test, were collected from Apple HealthKit. The standing metric recorded the number of hours each day during which the user stood and moved for at least one minute. Additionally, HealthKit provided an estimated six‐minute walk test result, calculated based on observed motion and workout data. This estimate aims to approximate the distance a user could achieve in a clinical six‐minute walk test, with a maximum distance estimate capped at 500 m. These parameters were averaged over the week to monitor patient progress post‐surgery. To ensure the accuracy and reliability of this data, the daily step counts were averaged on a weekly basis, thereby minimizing the impact of daily fluctuations or outliers. This weekly average was then monitored and recorded over the course of 1 year, providing a comprehensive and continuous assessment of the patient's activity levels throughout the post‐operative period. While tracking activity in terms of minutes was considered, it was not feasible with our current metrics, as they did not account for the duration of activity and could have been significantly influenced by short bursts of high activity. This methodology was chosen to deliver a more consistent and meaningful representation of the patients' mobility and recovery progress. Previous studies have confirmed the reliability and validity of the Apple Watch for these measurements [7, 29, 31].

### Post‐operative care

Post‐operative care following UKA involved a structured rehabilitation programme delivered through a digital application, allowing patients to engage in guided physical therapy from home. Patients were encouraged to begin weight‐bearing activities immediately after surgery, with the support of crutches as needed. The digital platform provided tailored exercises to improve strength, flexibility and range of motion, helping patients gradually transition to full weight‐bearing and independent mobility. Regular check‐ins through the app ensured adherence to the rehabilitation protocol and allowed for timely adjustments based on individual progress.

### Statistical analysis

Cohort characteristics and outcomes were summarized using descriptive statistics, including means, standard deviations (SDs) and frequencies. Given the study's sample size, the non‐parametric Wilcoxon signed‐rank test was used to assess the post‐operative differences in objective outcomes. Statistical analyses were performed using R software, version 4.2.3 and a significance level of *p* < 0.05 was considered statistically significant.

## RESULTS

### Patient‐reported outcomes measures

The study results show a consistent improvement in patient‐reported outcomes following UKA. The mean KOOS, JR increased from 50.73 (SD 11.32) preoperatively to 81.03 (SD 13.68) (*p* < 0.001) at one year post‐operatively, indicating significant enhancement in knee function and symptoms (Table 2). Notably, the most substantial improvement occurred within the first 3 months post‐surgery, with scores rising from 66.99 (SD 10.64) at 1 month to 74.48 (SD 15.34) at 3 months and continuing to improve through the 6‐month and 1‐year follow‐ups. Similarly, EQ‐5D scores, reflecting overall health‐related quality of life, showed improvement from a preoperative mean of 81.03 (SD 10.74) to 84.81 (SD 14.09) at 1 year (*p* < 0.001). The largest gain in EQ‐5D scores was observed 3 months post‐operatively, rising from 79.43 (SD 13.21) at 1 month to 85.25 (SD 12.65) at 3 months, with slight fluctuations noted at later time points.

**Table 2:** Patient‐reported outcome measures following unicompartmental knee arthroplasty.

Time point	KOOS, JR (mean (SD))	EQ‐5D (mean (SD))
Preoperative	50.73 (11.32)	81.03 (10.74)
1 Month post‐operative	66.99 (10.64)	79.43 (13.21)
3 Months post‐operative	74.48 (15.34)	85.25 (12.65)
6 Months post‐operative	77.62 (15.52)	85.79 (12.96)
1 Year post‐operative	81.03 (13.68)	84.81 (14.09)

Abbreviations: EQ‐5D, EuroQol‐5 Dimension; KOOS JR, Knee Injury and Osteoarthritis Outcome Score Joint Replacement.

### Daily steps

**Figure 1:**
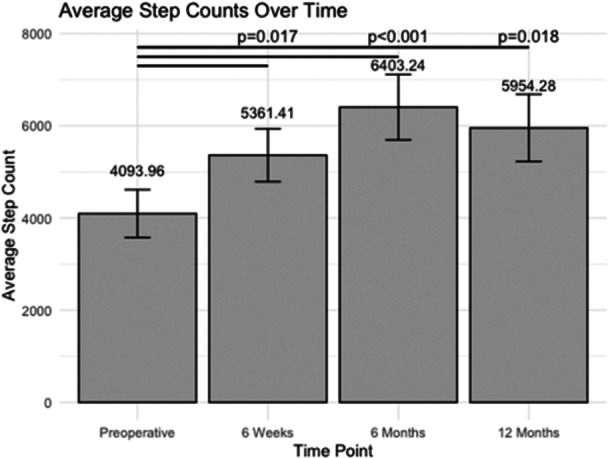
The average daily step count at various post‐operative time points following unicompartmental knee arthroplasty (number of steps per day).

During the preoperative period, participants recorded an average daily step count of 4094 ± 521 steps (Figure 1). Following surgery, there was a notable increase in the average daily step count compared to preoperative levels at 6 weeks (*p* = 0.017), 6 months (*p* < 0.001) and 12 months (*p* = 0.002) post‐operatively. At 6 months post‐operative, participants exhibited the highest average daily step count of 6403 ± 711 steps, followed closely by the 12‐month post‐operative period with an average of 5954 ± 730 steps per day.

### Steadiness

**Figure 2:**
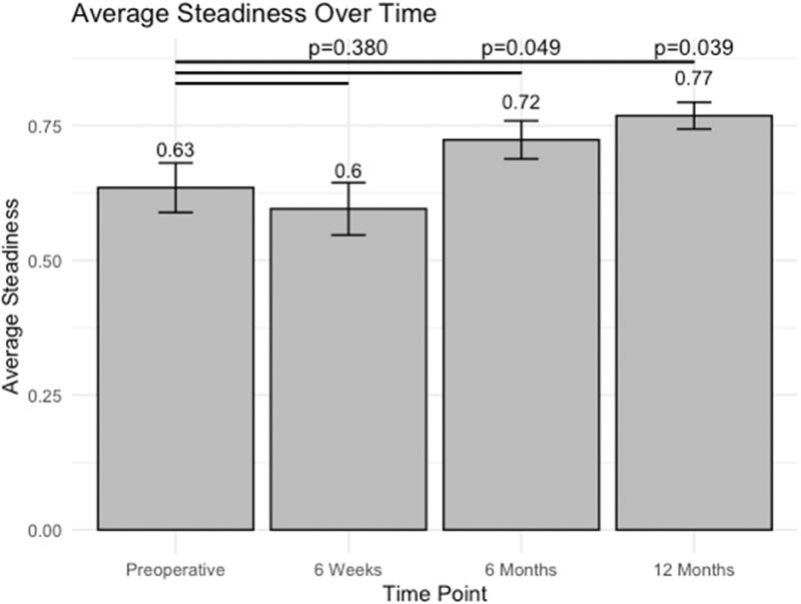
The average steadiness at various post‐operative time points following unicompartmental knee arthroplasty.

Before the operation, patients showed an average steadiness of 0.63 ± 0.05 (Figure 2). However, at 6 weeks post‐operative, there was a slight decrease in steadiness to 0.60 ± 0.05, although not statistically significant (*p* = 0.380). By the 6‐month post‐operative mark, patients demonstrated a significant improvement in steadiness, with a score of 0.72 ± 0.04, surpassing the preoperative level (*p* = 0.049). This improvement was sustained, with an average steadiness of 0.77 ± 0.02 at 12 months post‐operative (*p* = 0.039).

### Standing

**Figure 3:**
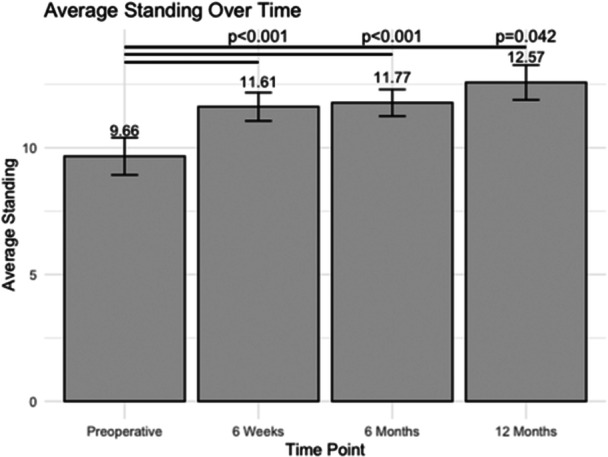
The average number of standing hours at various post‐operative time points following unicompartmental knee arthroplasty (number of hours per day).

At the preoperative time point, patients averaged 9.66 ± 0.74 h of standing per day (Figure 3). Following surgery, patients undergoing UKA showed a statistically significant increase in standing hours compared to the preoperative period, with 11.61 ± 0.56 h (*p* < 0.001), 11.77 ± 0.53 h (*p* < 0.001) and 12.57 ± 0.69 h (*p* = 0.042) at 6 weeks, 6 months and 12 months, respectively.

### Gait speed

**Figure 4:**
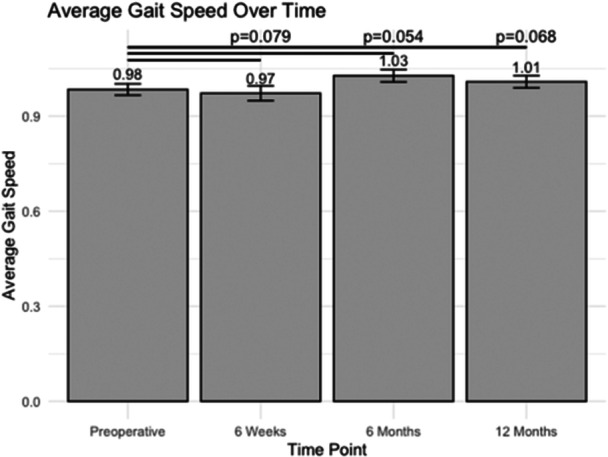
The average gait speed at various post‐operative time points following unicompartmental knee arthroplasty (m/s).

At the preoperative time point, the average gait speed was 0.98 ± 0.02 m/s (Figure 4). There were no significant changes in gait speed post‐operatively at any time point. At 6 weeks, 6 months and 12 months post‐operative, patients exhibited a gait speed of 0.97 ± 0.02 m/s (*p* = 0.079), 1.03 ± 0.02 m/s (*p* = 0.054) and 1.01 ± 0.02 m/s (*p* = 0.068), respectively.

### Estimated six‐minute walk test

**Figure 5:**
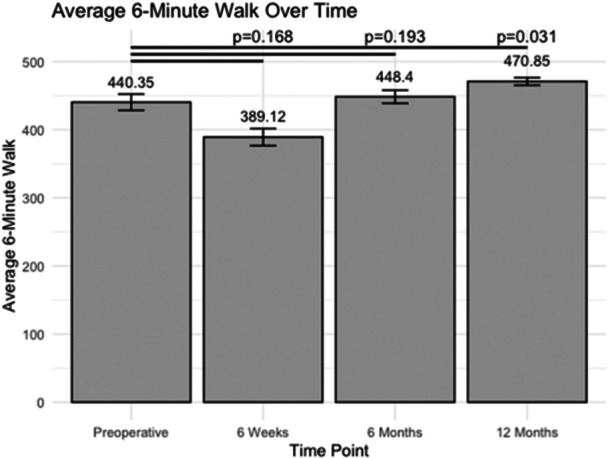
The average estimated six‐minute walk test at various post‐operative time points following unicompartmental knee arthroplasty (metres).

Before the surgery, the average estimated distance for the six‐minute walk test was 440.35 ± 11.93 m (Figure 5). Patients exhibited a similar estimated distance at the 6‐week post‐operative assessment, recording 360.07 ± 12.52 m (*p* = 0.168) and at the 6‐month mark, with 448.40 ± 9.62 m (*p* = 0.027). However, by the 12‐month follow‐up, patients achieved a significantly longer estimated distance for the six‐minute walk test, with 470.85 ± 5.58 m (*p* = 0.031).

## DISCUSSION

This pilot study aimed to evaluate changes in daily physical activity levels following UKA using objective metrics collected from wearable technologies. The results offer valuable insights into the functional recovery and post‐operative outcomes of patients undergoing UKA. The findings demonstrate a significant improvement in daily physical activity levels post‐operatively, as indicated by various objective measures. Factors such as step count, steadiness, standing time and estimated six‐minute walk test all showed significant increases at 12 months post‐operative compared to the preoperative phase, suggesting enhanced mobility and physical function in UKA patients. Similarly, previous research has demonstrated the improvements in mobility following UKA. Mullaji et al. reported that over 80% of their patients achieved greater than 130° of flexion following UKA, and a similar proportion of patients were able to perform activities such as sitting cross‐legged, kneeling, squatting and getting up from the floor following UKA [14]. The improvements in daily activity following UKA may be attributed to faster recovery of quadriceps muscle strength and improved gait, along with factors such as reduced soft‐tissue trauma, preservation of ACL and PCL function, and the selective replacement of only a single compartment in UKA [8, 9, 13, 20, 21, 22]. These improvements in muscle function may account for the significant increase in daily step count at 6 weeks, 6 months and 12 months post‐operatively compared to preoperative levels. In a study by Crizer et al., the daily step counts following total hip arthroplasty (THA) and TKA were observed at 12 weeks post‐operatively, with patients averaging 3884 and 2311 steps per day, respectively [5]. These findings, while showing improvement in mobility, highlight that the average step counts remain well below the World Health Organization's (WHO) recommended 10,000 steps per day for maintaining general health. It's important to recognize that patients undergoing joint replacement are often less active at baseline compared to individuals without OA. The chronic pain, stiffness and reduced joint function associated with OA can significantly limit their physical activity levels long before surgery. This lower baseline activity likely contributes to the difficulty in reaching the WHO's recommended 10,000 steps per day post‐operatively. Even with improvements in mobility following procedures like THA and TKA, these patients may continue to fall short of this target, underscoring the need for tailored rehabilitation programmes that take into account both their preoperative and post‐operative activity levels. This discrepancy likely reflects the inherent limitations faced by patients undergoing joint replacement, as these individuals may continue to experience discomfort, reduced functional capacity or other factors that limit their daily activity levels even after surgery. Understanding these limitations is crucial in setting realistic post‐operative recovery goals and tailoring rehabilitation programmes that gradually encourage increased physical activity while acknowledging the unique challenges faced by this patient population.

Steadiness, a metric of functional recovery, showed a slight decrease at 6 weeks post‐operatively, but significantly improved at 6 and 12 months compared to preoperative levels. Previous studies have noted the improvements in gait steadiness of UKA patients compared to TKA [3, 16, 25]. Jones et al. conducted a study on 12 patients who underwent UKA, assessing their gait at 12 months post‐operative [9]. They found that these patients exhibited a gait pattern close to normal physiology, but found that these improvements in gait were not reflected in the recorded Oxford knee scores. Our results suggest that patients have enhanced balance and stability in following UKA, which is crucial for daily activities and overall quality of life. The difference in steadiness is noted in the long term. Furthermore, our study can help inform patients that they may experience a decrease in steadiness during the acute phase of their recovery, justifying the need for gait aids in the acute post‐op period. Standing duration also increased significantly post‐operatively, with patients spending more hours standing per day at 6 weeks, 6 months and 12 months compared to preoperative levels. This increase reflects improved weight‐bearing capacity and functional ability in patients following UKA, which is essential for maintaining musculoskeletal health and preventing complications, as post‐operative falls remain a significant source of complications following joint replacement [27].

Gait speed, however, did not exhibit significant changes post‐operatively at any time point. While this might seem unexpected, it is important to consider that gait speed is influenced by various factors, such as pain, muscle strength and balance, which may not have been fully captured by our study's metrics. In patients where gait speed may be a concern, measures to reduce post‐operative knee swelling may be warranted to enhance quadriceps strength and gait speed [20]. The small sample size for this pilot study likely impacted our findings, as previous studies have suggested improvements in gait speed following UKA [25]. Alternatively, it is plausible that while patients can achieve faster speeds in a clinical testing setting, their day‐to‐day speed may not differ from preoperative levels. Previous studies have confirmed that functional ability is improved following UKA. Pongcharoen et al. conducted a randomized controlled trial comparing post‐operative recovery, specifically the two‐minute walk test and Timed Up‐and‐Go test, between 50 UKA and 49 TKA patients, finding faster recovery times in the UKA group [19]. Similarly, in our study, the estimated six‐minute walk test distance, which provides an indication of functional capacity and endurance, showed a significant improvement at the 6‐month and 12‐month follow‐up compared to preoperative levels. This improvement suggests that patients undergoing UKA experience enhanced functional capacity and endurance, even though their daily gait speed may be not as fast. Future research should further explore the relationship between daily activity and functional ability to provide a clearer understanding [24, 27].

Overall, the results of this pilot study support the hypothesis that objective measures of physical activity demonstrate improvements post‐operatively following UKA. These improvements reflect enhanced mobility, stability and functional capacity in patients undergoing UKA, highlighting the effectiveness of this surgical intervention in improving quality of life and functional outcomes in patients with knee OA. UKA may offer benefits that extend beyond mere improvements in functional scores, potentially leading to increased levels of daily physical activity. Despite previous research demonstrating general improvements in mobility and activity levels following UKA, our study provides novel insights by employing advanced wearable technologies to capture detailed, objective data on daily physical activity metrics. While earlier studies have affirmed the benefits of UKA in enhancing overall activity, our research delves deeper into specific metrics such as step count, steadiness, standing duration and six‐minute walk test performance. This fine‐grained analysis offers a more nuanced understanding of the recovery trajectory, and the functional improvements patients experience over time. By highlighting these detailed metrics, our study contributes to a richer comprehension of post‐operative outcomes, offering valuable data that can refine clinical practices and patient management strategies in ways that broader studies may not have addressed.

Limitations of this study include its small sample size and short follow‐up period, which may limit the generalizability of the findings. Given the observed effects in this study—significant improvements in daily step count, steadiness, standing duration and the six‐minute walk test—a post hoc power analysis was conducted to determine an appropriate sample size for future research. Assuming a significance level of 0.05 and aiming for a power of 0.80, it was estimated that a sample size of approximately 60–80 participants would be necessary to detect similar effect sizes with greater precision. This estimate accounts for the variability observed in the pilot study and is designed to ensure that future research is adequately powered to reliably assess the efficacy of UKA in enhancing daily physical activity and functional outcomes. Increased follow‐up is needed, as the recovery of patients may continue to improve beyond the one‐year mark, eventually reaching a point where they have an appropriate function. Felts et al. studied 62 UKA patients under the age of 60 and found that over 80.0% had resumed their sports activities on average 11 years following surgery, suggesting that more longitudinal research is needed [6]. Although we did not extend our follow‐up beyond 1 year, future research should aim to explore these long‐term outcomes to provide a more comprehensive understanding of the impact of UKA on patient mobility and overall quality of life. This would help to fully capture the trajectory of recovery and inform more effective post‐operative care strategies. Furthermore, the reliance on wearable technologies for data collection could introduce measurement errors or biases, potentially affecting result accuracy. The study's findings may not be fully generalizable to all UKA patients due to variations in demographics, surgical techniques and rehabilitation protocols.

Our study did not examine adverse events which could potentially have influenced the outcomes of this study. Additionally, we did not systematically track the use of assistive devices such as walkers or canes, which could have impacted the step counts observed. One possible explanation for the modest improvement in step count, with an average increase of around 2000 steps, could be related to several factors. According to the WHO, the recommendation is to achieve 10,000 steps per day for optimal health, yet the patients in our study, who are of working age, may have had limitations such as sedentary jobs or even disability pensions that could have affected their physical activity levels. These parameters, which were not fully captured in our study, might influence daily activity and should be considered in future research to provide a more comprehensive understanding of the factors affecting physical recovery and activity levels post‐surgery.

Future research should prioritize the analysis of relationships between patient‐reported outcome scores and these daily activity levels to gain a comprehensive understanding of how patients perceive their own health and quality of life, especially in relation to their daily activities. Our study serves as foundational work, providing an essential framework for examining how these subjective patient‐reported outcomes correlate with and are influenced by objective daily activity metrics. By building on our research, future studies can explore these relationships in greater depth, ultimately leading to more personalized and effective healthcare strategies that better align with patient experiences and needs. The lack of a control group, such as TKA, is another noted constraint. These factors may influence the generalizability of our findings. To address these limitations, future studies compare UKA against TKA, to provide a more comprehensive analysis of the outcomes and differences between these procedures. This may include incorporating data on clinical evaluations before and after surgery, as well as information on sports activity, to provide a more comprehensive understanding of patient outcomes following UKA. Additional research would enhance the contextual understanding of this study's findings and offer a more complete perspective on post‐operative activity levels and recovery trajectories.

## CONCLUSION

This pilot study provides valuable insights into the changes in daily physical activity levels following UKA. The results demonstrate significant improvements in various objective measures of physical activity post‐operatively, highlighting the benefits of UKA in improving mobility, stability and functional capacity in patients with knee OA. Understanding how daily activity levels change post‐surgery is crucial for counselling patients, as it provides realistic expectations for recovery and helps tailor rehabilitation programs to optimize their return to an active lifestyle. Further research with larger sample sizes and longer follow‐up periods is warranted to confirm these findings and explore the long‐term effects of UKA on physical activity and functional outcomes in patients with knee OA.

## AUTHOR CONTRIBUTIONS

All authors contributed to the idea, initiation, execution and revision of the study. Kevin A. Wu conceptualized the project, wrote the original draft and analyzed the data. Eric S. Dilbone, David N. Kugelman and Rahul K. Goel contributed to the data collection and revision of the draft. Sean P. Ryan, Samuel S. Wellman, Michael P. Bolognesi and Thorsten M. Seyler contributed to the patient data and supervised the project. All authors agreed on the order of authorship prior to manuscript submission. The authors read and approved the final manuscript.

## CONFLICT OF INTEREST STATEMENT

The authors declare no conflict of interest

## ETHICS STATEMENT

The study received approval from the institutional review board (IRB: Pro00115921) before the start.

## Data Availability

The data that support the findings of this study are available on request from the corresponding author. The data are not publicly available due to privacy or ethical restrictions.
